# Differentiation Between Agents and Patients in the Putative Two-Word Stage of Language Evolution

**DOI:** 10.3389/fpsyg.2021.684022

**Published:** 2021-08-11

**Authors:** Petar Gabrić

**Affiliations:** Institute for German Linguistics, Philipps University of Marburg, Marburg an der Lahn, Germany

**Keywords:** language evolution, semantic role, transitivity, cognitive evolution, semantic compositionality, word order, human evolution

## Introduction

Language evolution remains a hotly debated, yet somewhat controversial topic, due to our limited ability to experimentally investigate it and observe it in nature. While some researchers contend that modern-like language emerged in a single leap from a “languageless” state (Berwick, 1998; Chomsky, 2002; Berwick et al., 2013; Nóbrega and Miyagawa, 2015; Berwick and Chomsky, 2016, 2019; Chomsky et al., 2019; Tattersall, 2019; Reboul, 2021), others believe language evolution followed a more gradual path (Bickerton, 1990, 2000, 2007; Arbib, 2005; Hurford, 2007, 2012; Krause et al., 2007; Knight, 2009; Casielles and Progovac, 2012; Dediu and Levinson, 2013, 2014, 2018; McMahon and McMahon, 2013; Collier et al., 2014; Janković and Šojer, 2014; Tallerman, 2014, 2016; Lieberman, 2015; Everett, 2016; Planer, 2017; Gabrić et al., 2018, 2021; Michlich, 2018; Gabrić, 2019, 2021a,b; Progovac, 2019; Barham and Everett, 2020; Botha, 2020; Lameira and Call, 2020; Mounier et al., 2020; Neto, 2020). Several scholars from the latter school of thought have proposed that there was a two-word stage in the course of language evolution, in which utterances could not combine more than two words (Jackendoff, 1999; Gil, 2008, 2009; Hurford, 2012, p. 585ff.; Jackendoff and Wittenberg, 2014; Progovac, 2015, 2016; Benítez-Burraco and Progovac, 2020). These models agree that the putative two-word stage did not exhibit syntax. However, they disagree on whether or not there existed rules for inferring the semantic relationship between the two words expressing a compositional proposition. Focusing on semantically transitive events, I combine in the present paper language evolution models with previous empirical studies in linguistics to argue that the two-word stage was indeed governed by rules for inferring the compositional meaning of the utterance, in that (1) words were either associated with fixed (“predetermined”) semantic roles (i.e., agent, patient, predicate) or (2) there was a fixed order^1^ of semantic roles and the same words could be assigned different semantic roles in different utterances. Given the proposed existence of rules for producing and interpreting semantically compositional messages, it would appear that the putative two-word stage of language evolution did in fact exhibit syntax.

## One Word, Two Words…

Several accounts of language evolution have suggested that the first utterances had to be composed of only one word. Utterances comprised of a single denotative unit are found in wild non-human animals where they most often relate to vocalizations denoting predators and potentially intrusive species, as well as different food types (Struhsaker, 1967; Seyfarth et al., 1980; Karakashian et al., 1988; Cheney and Seyfarth, 1990; Evans et al., 1993; Uhlenbroek, 1996; Zuberbühler et al., 1999; Seddon et al., 2002; Crockford and Boesch, 2003; Brumm et al., 2005; Digweed et al., 2005; Slocombe and Zuberbühler, 2005; Egnor et al., 2006; Clay and Zuberbühler, 2009; Suzuki, 2012, 2016, 2019, 2020; Fischer, 2020; Snowdon, 2020). On the other hand, compositional utterances have only seldomly been documented in wild animals (Arnold and Zuberbühler, 2006, 2008, 2012; Ouattara et al., 2009a,b; Schlenker et al., 2016; Suzuki et al., 2017; Kuhn et al., 2018; Suzuki and Zuberbühler, 2019; Suzuki, 2021) and their status is disputed by some researchers, suggesting that the mere concatenation of (two) words to express a semantically compositional meaning may have been a paramount step in language evolution. Indeed, the currently undisputed (or little disputed) data on semantic compositionality in wild animals appear to be limited to cumulatively conjunctive meanings (i.e., “and”-meanings) (Boesch, 1991; Suzuki et al., 2016; Gabrić, 2021c). In other words, meaningful units constituting putatively compositional messages in wild animals are, according to current data, not assigned semantic roles similar to those in modern languages. Interestingly, all of the observed putatively compositional expressions in wild animal communication are limited to combinations of two units (“words”).

Gil (2009) and Progovac (2015, 2016), among others, have proposed that in the putative two-word stage, the semantic roles of the different words in a given utterance could not be readily inferred by the receiver, as per the lack of rules for understanding the compositionality of two-word utterances. Both Gil and Progovac use examples of semantically transitive events to make their point. Gil writes that in the Riau Indonesian sentence *Ayam makan*. “chicken eat,” it is not clear whether *ayam* “chicken” is the agent (the eater) or patient (the eaten)^2^. Similarly, following Minimalism, Progovac (2016) exemplifies this problem with the sentence *Deer eat*. Gil (2009) goes on to say that the receiver infers the compositional meaning of *Ayam makan*. via the so-called association operator which derives the general compositional meaning ENTITY ASSOCIATED WITH MEANING A AND MEANING B. Thus, both Gil and Progovac suppose that there were neither syntactic nor semantic rules for understanding two-word utterances but that, presumably, pragmatic processing was crucial in the early stages of language evolution.

## Rule 1: Words Associated With Fixed Semantic Roles

Let us stop and try to envisage a two-word stage of language evolution. Naturally, sentences (even two-word sentences) are semantically compositional, indicating the existence of at least some rule for coding and decoding the compositional message. In other words, the two words comprising a two-word sentence should each be associated with a specific semantic role. Intuitively, we can imagine either a language with a fixed order of the semantic roles assigned to the two words constituting the utterance (according to some rule) or a language with a free order of the semantic roles. In any case, the two words have to be in some semantic relationship and, in the case of semantically transitive scenarios, the words should express a particular semantic role in a given utterance such as agent, patient, predicate, etc. If the order of the semantic roles was free in such a language, this would mean (1) that in ~50% of the situations, the speakers would use this or that order for the same combination of words but (2) that the receivers would still understand the utterances in ~100% of the situations. If this is true, then the words could only be used with a fixed (“predetermined”) semantic role. In other words, a word such as *elephant* would be stored in the mental lexicon with a specified semantic role (agent or patient), alongside the word's phonological form and semantic content. A combination of two words such as *elephant* and *kill* could only convey either the proposition ELEPHANT KILL or ELEPHANT (BEING) KILLED, independently of whether the ELEPHANT-denoting word preceded or followed the KILL-denoting word. Otherwise, and in the absence of relevant situational stimuli (i.e., actually perceiving the elephant- and killing-related event), the receiver could never understand the compositional meaning (i.e., who did what to whom), despite understanding the individual words.

How plausible is it that words were associated with fixed semantic roles in the early stages of language evolution? Previous discussions on language evolution have already proposed that the earliest words must have had object-like and action-like meanings (somewhat corresponding to the morphosyntactic categories of protonouns and protoverbs) (Heine and Kuteva, 2007). This would suggest that there was at least one semantic type of words (action words) that would be associated with the semantic role of predicate. Furthermore, experimental studies have shown that nouns denoting animate concepts are more likely to express agents than patients in a given sentence, as well as that nouns denoting animate concepts in a sentential context are more likely to be interpreted by the receiver as an agent than a patient (Ferreira and Clifton, 1986; Garnsey et al., 1997; McRae et al., 1998). Still, these studies merely suggest that the semantic feature of animacy is associated with specific semantic roles and they do not suggest that specific words are associated with specific semantic roles. The same word may act as both the agent or patient in different sentences, even if the same verb is used, e.g., (cf. McRae et al., 1998, p. 284):

1. The cop arrested the thief.
2. The cop arrested by the FBI is innocent.

This might be especially true for words denoting concepts of social relationships (e.g., *leader, member, subordinate*, etc.), as well as names. It seems unlikely that such words had a fixed semantic role. However, given the relative abstract nature of the semantic content of such words (cf. Brysbaert et al., 2014), it remains questionable whether such words would have been present already in the two-word stage of language evolution. Nevertheless, the seeming existence of such concepts in wild chimpanzees and bonobos (as indicated by, e.g., the existence of alpha and beta males and other types of social relations in communities) renders this a possibility. There is, however, currently no evidence that such concepts are “lexicalized” in wild chimpanzees and bonobos.

Another possible issue with the proposed Rule 1 is that even though individual words might have been dominantly assigned one specific semantic role across contexts, this might not have been exclusive. In other words, the same words could have been assigned different semantic roles (albeit less frequently compared to the dominant role), with the distribution of the associations between individual words and assigned semantic roles being Zipfian in nature. If this was so, interspeaker variation in assigning semantic roles to individual words would have possibly relatively quickly (perhaps after several generations) led to a state where the same words could have been assigned different semantic roles, depending on the context.

## Rule 2: Fixed Order of Semantic Roles

Another possibility for the putative two-word stage of language evolution is that the order of the semantic roles assigned to the two words was somehow fixed. In some form, this has already been argued by Jackendoff and Wittenberg (2014, pp. 73–75) who proposed, based on an introspective analysis of compounds in English, that there might have been particular schemas founded on some kind of semantic relationship and that the semantic roles entailed in this relationship might have displayed a fixed order. One of their examples is the so-called modification schema in which the linearly first word would stand for the modifier, while the second would stand for the modified (e.g., *blackbird* = “bird that is black”). However, this and their other assumptions are based on a limited selection of examples of a morphosyntactic phenomenon in a particular language, while Jackendoff and Wittenberg don't pay much attention to the expression of transitive scenarios.

I propose that in an early language expressing semantically transitive events and having a fixed order of the semantic roles involved in semantically transitive events, there was an agent-first rule. There is a wealth of linguistic research suggesting that agents are a highly salient element in propositions in which they appear and that they are in many contexts associated with the first-word position in the sentence (Riesberg et al., 2019). Studies of typological variation of the basic word order have shown that the by far most prevalent word orders are subject-object-verb and subject-verb-object (41.03 and 35.44%^3^, respectively; Greenberg, 1963; Dryer, 2005; cf. Gell-Mann and Ruhlen, 2011; Kemmerer, 2012). Although the *subject* represents a syntactic category, while the *agent* represents a semantic category, in nominative-accusative languages there is often a convergence of the two, especially in the expression of semantically transitive events. Furthermore, SOV and SVO are the most common word orders in sign languages as well (Kimmelman, 2012; Napoli and Sutton-Spence, 2014), including “spontaneously” emerged sign languages such as the Al-Sayyid Bedouin Sign Language (Sandler et al., 2005). There is also a typological prevalence of nominative-accusative compared to ergative-absolutive languages (Nichols, 1993; Bickel et al., 2015). This is indeed to be expected because if we assume that the agent is cognitively the most salient component of a transitive event, we should predict that the agent (i.e., subject) is going to be morphologically coded in some neutral form (which is typically the case in nominative-accusative but not ergative-absolutive languages). Even when the subject (not necessarily agent) is not in the nominative case in nominative-accusative languages, such as in dative-nominative constructions in Icelandic and German (Barðdal et al., 2014), the first constituent may express an experiencer which is arguably the closest semantic role to the agent, e.g.:

(3) **Mir** gefällt das.
I\DAT like.3sg that.
“I like that.” (lit. Me likes that.)

Further evidence comes from neurophysiological studies demonstrating that the first noun phrase in a given sentence is automatically interpreted as the subject and/or agent via specific neural mechanisms. Bornkessel et al. (2004) conducted an ERP study in which they investigated the reception of dependent object clauses in which the syntactical roles of subject and dative object, i.e., semantic roles of agent and target, were ambiguous until the last word in the sentence whose place was taken by a finite form of the auxiliary verb for building the perfect tense, e.g.,:

(4) … dass Betram Surferinnen gratuliert hat.
  “… that Betram congratulated the [female] surfers.”
(5) … dass Betram Surferinnen gratuliert haben.
  “… that the [female] surfers congratulated Betram.”

In the type of clauses as in (5), the authors observed in the evoked brain potentials a combination of biphasic negativity after 400 ms and late positivity. No significant amplitudes were observed in the first type of clauses. Thus, the first noun phrase is automatically analyzed as a subject and/or agent until there is a “rule break” (i.e., an unexpected grammatical phenomenon) and the sequence is re-analyzed. Similar results using ERP have been obtained by Bickel et al. (2015) who studied this in Hindi, a language displaying ergativity in some contexts. Agent saliency is observed in non-verbal contexts as well. In a range of studies, it has been shown that during the processing of visual stimuli depicting semantically transitive events, subjects who have prior information about the agent better predict the possible actions compared to subjects who have prior information about the patient, that agents are viewed longer compared to patients independently of the order of presentation, and that visual depictions of events are processed faster after agent presentation compared to patient presentation (Cohn and Paczynski, 2013; Cohn et al., 2017).

## Discussion

In conclusion, I disagree with Gil's (2009) and Progovac's (2015, 2016) proposals that the semantic roles of the two words constituting sentences in the putative two-word stage of language evolution were ambiguous to the receiver. I have proposed based on introspection and published empirical data two possibilities how agents and patients might have been inferred from two-word utterances expressing transitive events. Future research could investigate the degrees of modern humans' sensitivity to variations in the order of semantic roles during learning or creating artificial languages.

Nevertheless, there are important limitations to this opinion paper as well. Firstly, as pointed out by the reviewer, the communication between speakers need not have ended after the two-word sentence had been produced. The same speaker may have continued producing one- or two-word utterances (as per the definition of the two-word stage), thus opening up the possibility that semantically transitive events were coded using multiple sentences and not only a single two-word sentence. This is especially interesting given that semantically transitive events by their nature involve at least three phenomena: the agent, patient, and action. Future discussions on language evolution should consider this possibility. Secondly, although the processing of semantically transitive meanings has been found to be embodied in the sensorimotor system (Glenberg and Kaschak, 2002; Hauk et al., 2004; Tettamanti et al., 2005; Aziz-Zadeh et al., 2006; Desai et al., 2010; Kemmerer, 2012; Scorolli et al., 2012; Ghio and Tettamanti, 2016; Grisoni et al., 2016; Mollo et al., 2016; van Dam and Desai, 2016; Progovac et al., 2018), arguably suggesting the reuse of phylogenetically relatively ancient processing systems, it is by no means straightforward that the first two-word utterances expressed transitive propositions. In fact, and as already discussed by Jackendoff and Wittenberg (2014), other semantic combinations are imaginable as well, while some are documented in wild non-human animals (e.g., cumulative conjunction). Nevertheless, the proposed linguistic universality of some aspects of both semantic and syntactic transitivity (Creissels, 2016) suggests the possibility that the expression of transitive propositions was present in the early stages of language evolution.

## Author Contributions

The author confirms being the sole contributor of this work and has approved it for publication.

## Funding

Open Access funding was enabled by the Publications Fund of the University Library of the Philipps University of Marburg. No funds, grants, or other support were received.

## Conflict of Interest

The author declares that the research was conducted in the absence of any commercial or financial relationships that could be construed as a potential conflict of interest.

## Publisher's Note

All claims expressed in this article are solely those of the authors and do not necessarily represent those of their affiliated organizations, or those of the publisher, the editors and the reviewers. Any product that may be evaluated in this article, or claim that may be made by its manufacturer, is not guaranteed or endorsed by the publisher.

## Footnotes

^1^In the present paper, I use the not so widespread term *order of semantic roles* as opposed to *order of syntactic constituents* or, more loosely, *word order*. Typically, (basic) word order refers to the order of syntactic constituents (e.g., subject, object, predicate, etc.), independently of which semantic roles have been assigned to the words/phrases realized as particular syntactic constituents (Newmeyer, 2003). It is important to note that particular semantic roles do not have to correspond to particular syntactic constituents, even though there is not seldomly a convergence of some, e.g., of the subject and agent (e.g., Chan et al., 2009; Shimada et al., 2020; Ünal et al., 2021).

^2^A year ago I asked an Indonesian waiter in a restaurant in Cologne what *Ayam makan*. means and he replied *Chicken eats*. A similar experience is reported by Hurford (2012, p. 406).

^3^When languages with no apparent basic word order (13.73%) are counted as well.
