# Corrigendum: Differentiation Between Agents and Patients in the Putative Two-Word Stage of Language Evolution

**DOI:** 10.3389/fpsyg.2021.837686

**Published:** 2022-01-17

**Authors:** Petar Gabrić

**Affiliations:** Institute for German Linguistics, Philipps University of Marburg, Marburg an der Lahn, Germany

**Keywords:** language evolution, semantic role, transitivity, cognitive evolution, semantic compositionality, word order, human evolution

In the original article, no funding information was reported. The corrected funding statement is shown below.

Open Access funding was enabled by the Publications Fund of the University Library of the Philipps University of Marburg. No funds, grants, or other support were received.

The author apologizes for this error and states that this does not change the scientific conclusions of the article in any way. The original article has been updated.

## Publisher's Note

All claims expressed in this article are solely those of the authors and do not necessarily represent those of their affiliated organizations, or those of the publisher, the editors and the reviewers. Any product that may be evaluated in this article, or claim that may be made by its manufacturer, is not guaranteed or endorsed by the publisher.

